# Whose Gene Is It Anyway? The Effect of Preparation Purity on Neutrophil Transcriptome Studies

**DOI:** 10.1371/journal.pone.0138982

**Published:** 2015-09-24

**Authors:** Huw B. Thomas, Robert J. Moots, Steven W. Edwards, Helen L. Wright

**Affiliations:** 1 Institute of Integrative Biology, University of Liverpool, Liverpool, United Kingdom; 2 Institute of Ageing and Chronic Disease, University Hospital Aintree, University of Liverpool, Liverpool, United Kingdom; Louisiana State University, UNITED STATES

## Abstract

Protocols for the isolation of neutrophils from whole blood often result in neutrophil preparations containing low numbers (~5%) of contaminating leukocytes, and it is possible that these contaminating cells contribute to highly sensitive assays that measure neutrophil gene expression (e.g. qPCR). We investigated the contribution of contaminating leukocytes on the transcriptome profile of human neutrophils following stimulation with inflammatory cytokines (GM-CSF, TNFα), using RNA-Seq. Neutrophils were isolated using Polymorphprep or the StemCell untouched neutrophil isolation kit (negative selection of “highly pure” neutrophils). The level of contamination was assessed by morphology and flow cytometry. The major source of contamination in Polymorphprep neutrophil preparations was from eosinophils and was highly donor dependent. Contaminating cells were largely, but not completely, absent in neutrophil suspensions prepared using negative selection, but the overall yield of neutrophils was decreased by around 50%. RNA-seq analysis identified only 25 genes that were significantly differentially-expressed between Polymorphprep and negatively-selected neutrophils across all three treatment groups (untreated, GM-CSF, TNFα). The expression levels of 34 cytokines/chemokines both before and after GM-CSF or TNFα treatment were not significantly different between neutrophil isolation methods and therefore not affected by contributions from non-neutrophil cell types. This work demonstrates that low numbers (<5%) of contaminating leukocytes in neutrophil preparations contribute very little to the overall gene expression profile of cytokine-stimulated neutrophils, and that protocols for the isolation of highly pure neutrophils result in significantly lower yields of cells which may hinder investigations where large numbers of cells are required or where volumes of blood are limited.

## Introduction

Neutrophils respond to, and influence, immune signalling between several different cell types, acting as a signalling bridge between the innate and adaptive immune systems[1]. Indeed, they are central to the progression of the immune response and can shape the outcome of inflammation or infection in response to the signals they receive from the local environment[2,3]. Given the growing appreciation of the importance of cell:cell signalling in neutrophil function and activity, it is perhaps somewhat paradoxical that the majority of *in vitro* studies use neutrophil preparations that are minimally-contaminated with other leukocytes, but the contribution of these contaminating cells to the overall assay output is often ignored. Neutrophil isolation techniques generally achieve a final neutrophil purity of >95% and viability of >97%. Isolation media such as Ficoll-Paque and Polymorphprep exploit the differences in size and density of different blood cells to separate cells into distinct populations. These methods require multiple centrifugation steps and overall isolation times of 60–90 min. Antibody-based techniques dramatically decrease the number of centrifugation steps required and achieve a higher final purity of cells (>99%), often in a similar time frame to density-gradient techniques. However, the higher purity afforded by these techniques usually comes at a greater financial cost and lower yield[4,5]. An additional concern when choosing an antibody-based isolation method is whether it utilises positive or negative selection. Isolation of neutrophils by positive selection (e.g. CD15 or CD16b) carries an increased risk of inadvertently changing their phenotype and most importantly, their functional capacity[6]. Alternatively, negative selection approaches require a mixture of specific antibodies that recognise cell surface markers on other immune cell types, which can significantly increase the cost and efficiency of the methodology but can lead to highly-pure, non-activated neutrophils. An additional consideration when isolating neutrophils is their relative sensitivity to physical stimuli that may promote apoptosis in the absence of stimulating factors; neutrophils are easily activated by shear forces, over-agitation, or centrifugation. It is therefore of equal importance that the suspension of neutrophils obtained from a purification protocol contains both high numbers of viable cells and low numbers of contaminating leukocytes.

The low percentage (<5%) of contaminating cells present in neutrophil suspensions following density-gradient isolation methods is often considered to be an acceptable level of contamination that has minimal effects on the behaviour of neutrophils in the preparations. However, the impact of contaminating leukocytes in neutrophil suspensions has been questioned; for example, Sabroe and colleagues demonstrated that the anti-apoptotic effect of lipopolysaccharide on ultra-pure neutrophils (>99%) was significantly decreased compared to neutrophils with 5% PBMC contamination[7,8]. Whilst it is commonly assumed that contaminating monocytes in neutrophil preparations may affect interpretation of data, it is often other granulocytes (in particular eosinophils) that constitute the largest proportion of non-neutrophil cells in most neutrophil preparations. Since neutrophils, eosinophils and basophils have very similar size and density, it is not possible to separate them from each other using approaches based on density-gradient media. The level of leukocyte contamination is usually donor-dependent, and is often also reliant on the technical dexterity of the researcher. It was recently shown that the percentage of contaminating cells in neutrophil preparations isolated by Polymorphprep varied between 1–17% across 18 individual blood samples from healthy controls.[9] These levels of contamination are of potential concern when using high-sensitivity technologies such as qPCR, mass spectrometry or RNA-Seq. Indeed, it has been suggested that eosinophils have a far greater transcriptional capacity than neutrophils, confounding experiments that quantify mRNA in neutrophil suspensions containing high and variable numbers of eosinophils[10].

We previously demonstrated that RNA-seq is a well-validated protocol to measure the neutrophil transcriptome, which can accurately measure gene expression and predict the functional phenotype of these cells[11,12]. The aim of this research was to quantify the differences in gene expression profiles of ultra-pure (>99%) neutrophils compared to neutrophils isolated using density gradient centrifugation, and to evaluate the effects of contamination by non-neutrophil cells on the neutrophil response to inflammatory cytokines. We show that whilst the main cellular contaminants in neutrophil isolations are eosinophils, they have minimal effect on the overall transcriptome profile in response to inflammatory cytokines, or the number of differentially expressed genes. In addition, we show that a significant proportion of neutrophils are lost during negative selection.

## Methods

### Neutrophil isolation using density gradient centrifugation

Ethical approval for the study of neutrophils from adult healthy controls was granted by the University of Liverpool Committee for Research Ethics. All participants gave written, informed consent. Blood was collected by venupuncture into lithium heparin-coated vacutainers and processed immediately. Neutrophils were isolated by single-step centrifugation of whole blood onto Polymorphprep (Axis-Shield) as per the manufacturer’s recommendation. Briefly, whole blood was layered onto Polymorphprep at a ratio of 1:1 and centrifuged at 500g for 35 min. The granulocyte layer was carefully removed and resuspended in RPMI 1640 media plus 25mM HEPES (Gibco) and centrifuged at 500g for 5 min to remove any remaining Polymorphprep. Cells were resuspended in media and contaminating erythrocytes were removed by hypotonic lysis. Platelet contamination was removed by further centrifugation of the cells at 150g for 3 min. Neutrophils were resuspended in RPMI 1640 media at a final concentration of 5x10^6^/mL.

### Neutrophil isolation using negative selection

The EasySep Human Neutrophil enrichment kit (StemCell) was used, following the manufacturer’s instructions. Briefly, whole blood was mixed with HetaSep solution (StemCell) at a ratio of 1:5 (HetaSep: whole blood) and incubated at 37°C for 30 min until the plasma/erythrocyte interphase was at approximately 50% of the total volume. The leukocyte-rich plasma layer was carefully removed and washed in a 4-fold volume of recommended media (Mg^2+^ and Ca^2+^-free PBS, + 2% FBS and 1mM EDTA). Cells were centrifuged at 500g for 5 min and resuspended in a 4-fold volume of recommended media and then at 120g for 10 min to remove platelet contamination and finally resuspended at 5x10^7^ nucleated cells/mL. 50μL of EasySep^®^ neutrophil enrichment cocktail, containing a mix of tetrameric antibody complexes produced from monoclonal antibodies (also bispecific for dextran) directed against the cell surface antigens CD2, CD3, CD9, CD19, CD36, CD56 and glycophorin A was added per 1mL of nucleated cells and incubated for 10 min at room temperature. 100μL of EasySep dextran-coated nanoparticle beads were added per 1mL of nucleated cells and incubated for a further 10 min at room temperature. The cell/antibody/bead solution was adjusted to a total volume of 2.5mL with recommended media and placed into an EasySep magnet for 5 min at room temperature. Unbound neutrophils were decanted and placed into an EasySep magnet for a further 5 min. Highly-pure, unbound neutrophils were briefly centrifuged and resuspended in RPMI 1640 media plus 25mM HEPES to a concentration of 5x10^6^/mL. In some experiments, washed leukocytes from the leukocyte-rich plasma layer were layered onto Ficoll-Paque (GE Healthcare) 1:1 and centrifuged at 500g for 30 min. The granulocyte pellet was resuspended in recommended media, centrifuged for 3 min at 500g and resuspended in recommended media. Granulocytes were quantified prior to neutrophil enrichment using the EasySep Human Neutrophil enrichment kit.

### Quantification of neutrophil purity

Purity of neutrophil preparations was determined by morphology of stained cytospins and flow cytometry. For cytospins, 10^5^ cells (in PBS with 10mM EDTA) were centrifuged onto a glass slide at 30*g* for 5 min using a Shannon 3 cytospin and immediately stained with Rapid Rowmanowsky stain (HD Supplies). For the measurement of cell surface markers by flow cytometry, 10^5^ cells were resuspended in PBS (+0.2% BSA) and incubated at 4°C in the dark with 2–5μL of fluorescently-conjugated antibody (CD11b-FITC, R&D Systems; CD15-FITC, Dako; CD16-FITC, BD Biosciences; CD64-FITC, R&D Systems; isotype control-FITC, Santa Cruz) for 30 min. Cells were fixed in 2% paraformaldyhyde prior to analysis. A minimum of 5000 gated events were analysed using a Guava EasyCyte flow cytometer (Miltenyi Biotech).

### Measurement of gene expression by RNA-Seq

For these experiments, two healthy donors were selected, based on the levels of non-neutrophil leukocytes that consistently contaminated neutrophil preparations obtained following isolation on Polymorphprep; Donor 1 had consistently low levels of contaminating cells (~1–5% by cytospin), whereas Donor 2 had consistently high levels of contaminating cells (~10–18% by cytospin) which were, in the main, eosinophils. Both donors were otherwise healthy and of similar age. Following isolation, neutrophils were incubated at 5x10^6^/mL for 1h in the absence or presence of TNFα (10ng/mL, Calbiochem) or GM-CSF (5ng/mL, R&D systems). RNA was isolated using TRIzol-chloroform (Invitrogen) precipitation as per the manufacturer’s protocol. The RNA precipitate was cleaned up using an RNeasy mini kit (Qiagen), which included a DNAse digestion step. Total RNA concentration and integrity were assessed using the Agilent 2100 Bioanalyser RNA Nano chip (Bio-Rad). RNA integrity (RIN) was routinely found to be ≥7.0. Total RNA was enriched for mRNA using poly-A selection, and standard Illumina protocols were used to generate 50bp single-end read libraries. Libraries were sequenced on the Illumina HiSeq 2000 platform. Reads were mapped to the human genome (hg19) using TopHat v2.0.4[13], and gene expression values were calculated using Cufflinks v2.0.2[14]. Calculating gene expression using Cufflinks produces the expression metric, reads per kilobase of exon model per million mapped reads (RPKM) which is calculated as (number of mapped reads x 1 kilobase x 1 million mapped reads) / (length of transcript x number of total reads). Therefore, in addition to normalising to the total number of reads in each sample, RPKM is also normalised to the size of the transcript. This means that expression values for two genes within the same sample can be compared. Equally, expression of a single gene in multiple separate experiments can be compared. A minimum RPKM threshold of expression of ≥0.3 was applied to the data in order to minimise the risk of including false positives against discarding true positives from the dataset[11,15]. Differential expression analysis was carried out using Cufflinks v2.0.2. A 5% false discovery rate (FDR) correction (Benjamini-Hochberg) was applied to the p-value (FDR-adjusted q-value) and a significance cut-off of FDR≤0.05 was used to select genes with significantly differential expression. Bioinformatics analysis was carried out using IPA (Ingenuity® Systems, www.ingenuity.com). Hierarchical cluster analysis was carried out using MeV[16] with Euclidean clustering and average linking. The data reported in this manuscript have been deposited in the Gene Expression Omnibus (GEO)[17] and are accessible through GEO Series accession number GSE70068.

### Statistical analysis

Statistical analysis was carried out using paired Student’s t-test and Pearson correlation unless otherwise stated.

## Results

### Quantification neutrophil purity following isolation

In our laboratory, isolation of neutrophils from herparinised whole blood using Polymorphprep routinely obtains preparations with >95% purity; contaminating cells are mainly eosinophils with <1% PBMCs (data not shown). For these studies, two healthy donors were selected, based on the levels of non-neutrophil leukocytes that consistently contaminated neutrophil preparations obtained following isolation on Polymorphprep; Donor 1 had consistently low levels of contaminating cells (~1–5% by cytospin), whereas Donor 2 had consistently high levels of contaminating cells (~10–18% by cytospin) which were, in the main, eosinophils. Both donors were otherwise healthy and of similar age.

**Fig 1:**
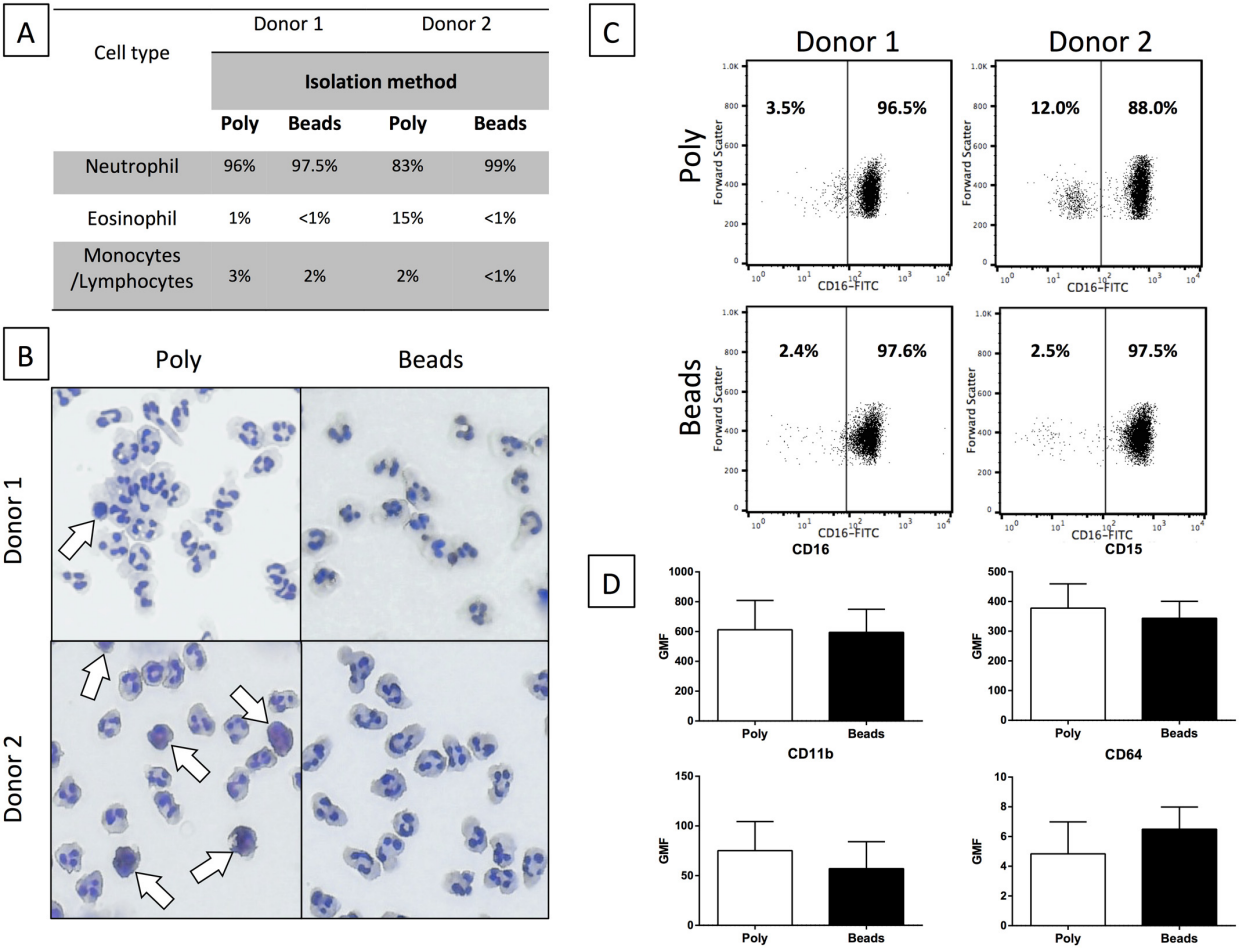
Neutrophil purity after isolation using Polymorphprep or negative-selection (magnetic beads). (A) Percentage of leukocytes in each preparation from each donor. Cells quantified by cell morphology and staining properties using cytospins (calculated from 4 separate fields of view, counting > 100 cells per field per donor). (B) Representative cytospins of neutrophil preparations following Polymorphprep (Poly) or negative selection (Beads) isolation protocols from Donor 1 (top) and Donor 2 (bottom). White arrows highlight non-neutrophil cells. (C) Flow cytometry scatterplots of neutrophil preparations by Polymorphprep (Poly) or negative selection (Beads) isolation protocols. Plotted by green fluorescence (CD16 positive, X-axis) and forward-scatter (Y-axis). Donor 1 (left panels) and Donor 2 (right panels). Numbers shown are percentage of cells in each of the two quadrants shown. (D) Levels of expression of cell surface markers in neutrophils isolated by Polymorphprep (Poly) or by negative selection (Beads). Geometric mean fluorescence (GMF) of CD16 (N = 5), CD15 (N = 3), CD11b (N = 3) and CD64 (N = 4) was measured by flow cytometry and normalised to an appropriate isotype control. Error bars represent SEM.

The levels of contamination of neutrophil preparations following Polymorphprep isolation or negative selection from the blood of Donors 1 and 2 were quantified by cytospin and flow cytometry. Cytospins were prepared and the mean number of neutrophils, monocytes, lymphocytes and eosinophils quantified in four separate fields of view (x20 magnification). A minimum of 100 cells were counted in each field (Fig 1A and 1B). Similar levels of lymphocyte and monocyte contamination were seen in both donors (Fig 1A and 1B). However, the greatest differences between donors was the number of eosinophils following Polymorphprep isolation, with Donor 1 having 1% eosinophils and Donor 2 having 15% eosinophils (Fig 1A and 1B). The overall purity of neutrophils was higher following negative selection than with Polymorphprep (Donor 1: 96% Polymorphprep, 97.5% negative selection; Donor 2: 83% Polymorphprep, 99% negative selection). Morphological analysis of levels of apoptosis immediately after isolation revealed that neither isolation method resulted in significant levels of apoptotic neutrophils (<1%, data not shown).

Eosinophils are difficult to distinguish from neutrophils by analysis of their forward-scatter and side-scatter profiles using flow cytometry[18,19] due to their similar sizes and granularity. However, they can be distinguished from neutrophils based on their levels of auto-fluorescence or cell surface expression levels of CD16 (FcγRIII); eosinophils have a higher autofluorescence and express lower levels of CD16 on their cell surface than neutrophils. Neutrophil suspensions were stained with FITC-conjugated anti-CD16 monoclonal antibody and analysed by flow cytometry. Cells were first gated on their forward- and side-scatter profiles so that subsequent measurements were made on granulocytic cells. Gated cells were analysed by their forward-scatter and CD16 properties (Fig 1C). Two distinct populations were detected: cells which were CD16^bright^ and cells of similar size which were CD16^dim^. These populations represent neutrophils and eosinophils, respectively. Quantification of the eosinophil population confirmed that neutrophils isolated by Polymorphprep contained a higher proportion of eosinophils than neutrophils isolated by negative selection. Donor 1 had 3.5% eosinophil contamination following Polymorphprep isolation, compared to 2.42% by negative selection, whereas Donor 2 had 12% eosinophil contamination following Polymorphprep separation, compared to 2.5% following negative selection. These levels of eosinophil contamination correlate well with the levels of purity as assessed by cytospins (Fig 1A).

To further assess levels of neutrophil purity and activation following Polymorphprep or negative selection, freshly-isolated neutrophils were stained with FITC-conjugated-monoclonal antibodies for CD16b (FcγIIIb), CD15 (3-fucosyl-N-acetyl-lactosamine), CD11b (Mac-1α) and CD64 (FcγRI) and relative fluorescence measured by flow-cytometry (Fig 1D). Levels of CD16, CD15 and CD11b were slightly lower in neutrophils isolated by negative selection whilst levels of CD64 were slightly lower in Polymorphprep isolated neutrophils. However, these differences were not significantly different (p>0.05) suggesting that neutrophil isolation methods and preparation purity have only marginal effects on cell surface receptor expression.

### Neutrophil yield from whole blood

**Fig 2:**
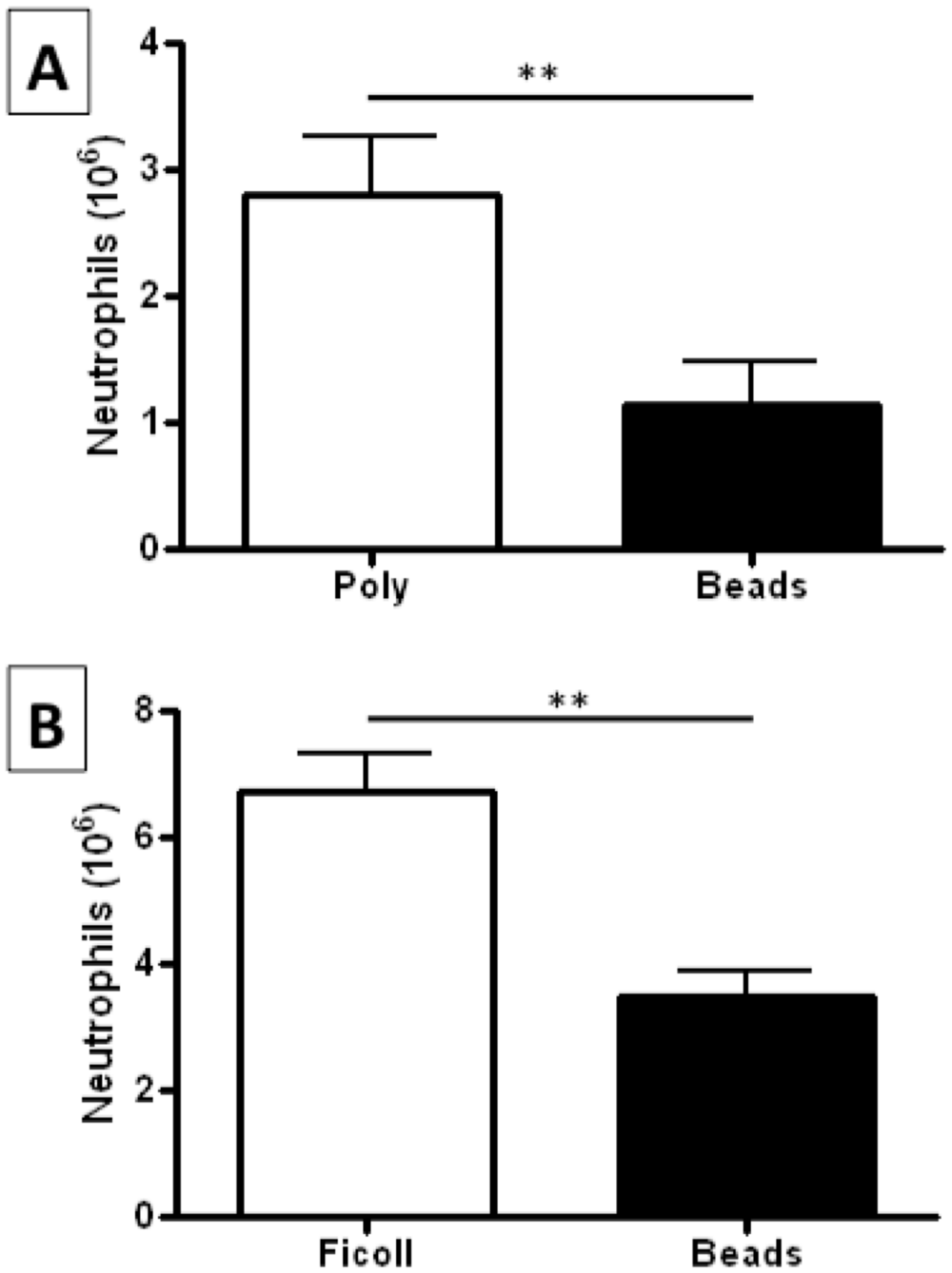
Neutrophil yield using density gradient isolation or negative selection. (A) Neutrophil yield (10^6^/mL) from whole blood isolated by Polymorphprep and negative selection (Beads). Data represents n = 5 paired neutrophil isolations (**p<0.01). (B) Neutrophil yield (10^6^/mL) from whole blood isolated by Ficoll-Paque (Ficoll) and negative selection (Beads). Data represent n = 5 experiments in which neutrophil enrichment from the Ficoll-Paque granulocyte pellet was carried out using negative selection (**p<0.01).

In addition to purity levels, different isolation methods can yield significantly different numbers of neutrophils[4,5]. To quantify differences between Polymorphprep and negative selection of neutrophils, samples of whole blood from healthy donors (n = 5) were divided into two aliquots, and subjected to both methods of neutrophil isolation. Final preparations of neutrophils were counted using a coulter counter and the number of neutrophils recovered per mL of whole blood was calculated. Mean numbers of neutrophils obtained after negative selection were only ~40% of those recovered after Polymorphprep isolation using blood from the same donor (Fig 2A, p<0.01). This suggests that a large proportion of whole blood neutrophils are somehow “lost”during negative selection. The first step of the negative selection protocol is an osmotic depletion of erythrocytes by aggregation and sedimentation under atmospheric pressure. This typically produces a leukocyte-rich plasma layer which is ~50% of the initial volume of whole blood, which is subsequently used in the negative selection isolation. In order to investigate whether this may account for the number of neutrophils “lost” in the negative selection protocol, neutrophils were first isolated from whole blood of healthy donors (n = 5) using Ficoll-Paque. The number of cells in the granulocyte pellet were quantified and found to be ~95% neutrophils (data not shown). Highly-pure neutrophils were then enriched from the granulocyte pellet using negative selection. We again observed that ~50% of neutrophils were “lost” during the negative selection protocol (Fig 2B, p<0.01) suggesting that a population of neutrophils are being retained by the antibody:bead complex used in the negative selection protocol.

### RNA-Seq analysis of neutrophils isolated by Polymorphprep and negative selection

**Fig 3:**
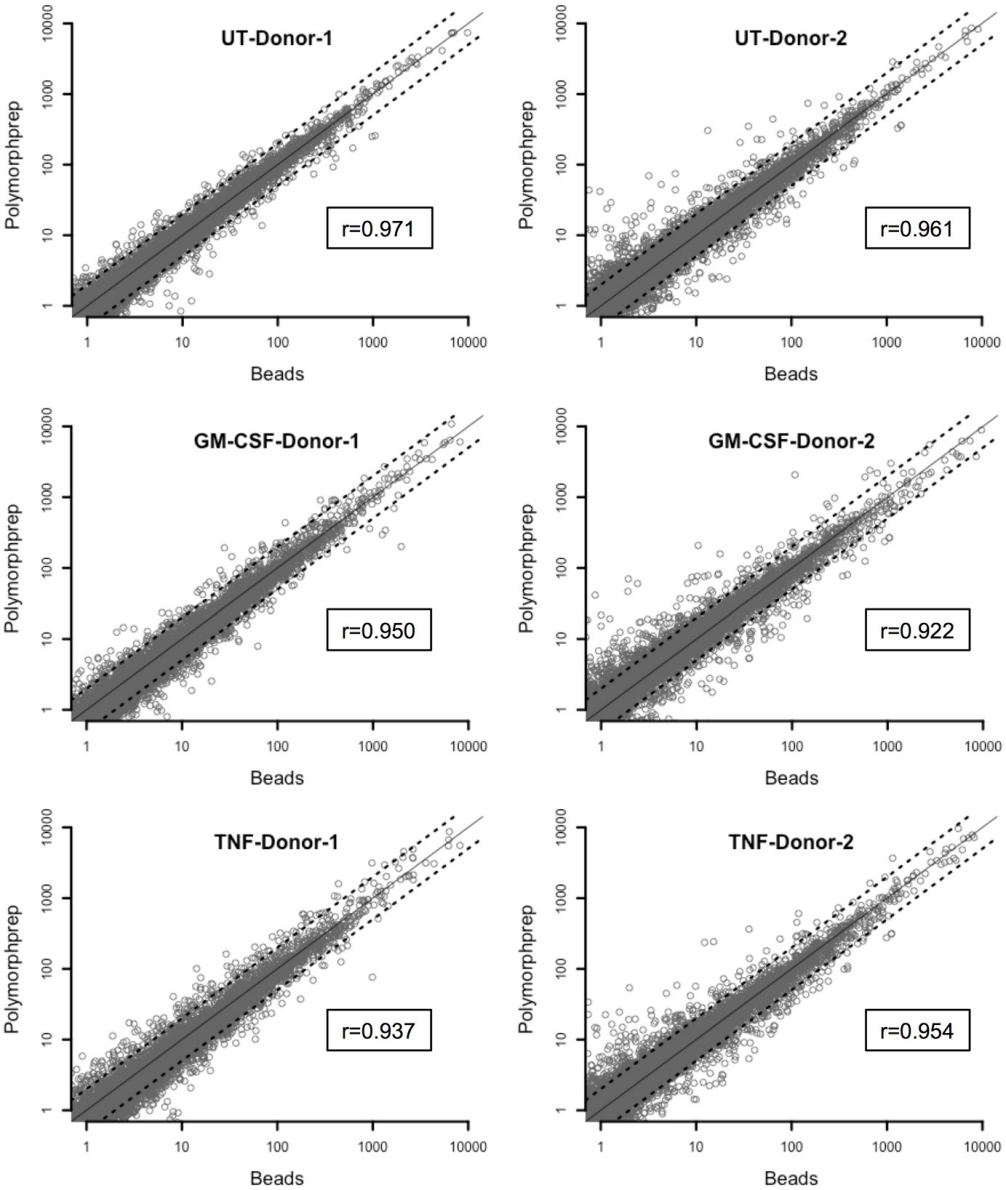
Correlation between transcriptomes of Polymorphprep and negatively-selected neutrophils. Gene expression (RPKM) in neutrophils isolated by Polymorphprep or negative selection (Beads) for Donors 1 and 2 following incubation with or without the addition of GM-CSF (5ng/mL) or TNFα (10ng/mL) for 1h. Pearson Correlation co-efficient is shown (p<0.01). The solid line indicates a correlation coefficient of r = 1.0 and the dashed lines indicate a fold difference in expression of 2.

Having shown that different donors and different neutrophil isolation procedures generate suspensions containing different numbers of contaminating cells, notably eosinophils, it was then necessary to determine how these contaminating cells and isolation techniques contribute to transcriptome studies. Neutrophils were isolated from Donor 1 and Donor 2 blood samples by Polymorphprep and negative selection, and incubated with (or without) inflammatory cytokines (GM-CSF or TNFα) for 1h. RNA was extracted and gene expression quantified using RNA-Seq. Gene expression values (RPKM) across the whole transcriptome correlated significantly between paired neutrophil samples prepared by the two methods (Polymorphprep and negative selection) from each donor, under all three experimental conditions (1h untreated, GM-CSF, TNF) (Fig 3, Pearson Correlation coefficient r is shown for each condition, p<0.01). More outlying transcripts were found in neutrophils from Donor 2, of which several corresponded to eosinophil-specific transcripts, such as CLC, which were expressed at higher levels in the preparation with Polymorphprep.

**Fig 4:**
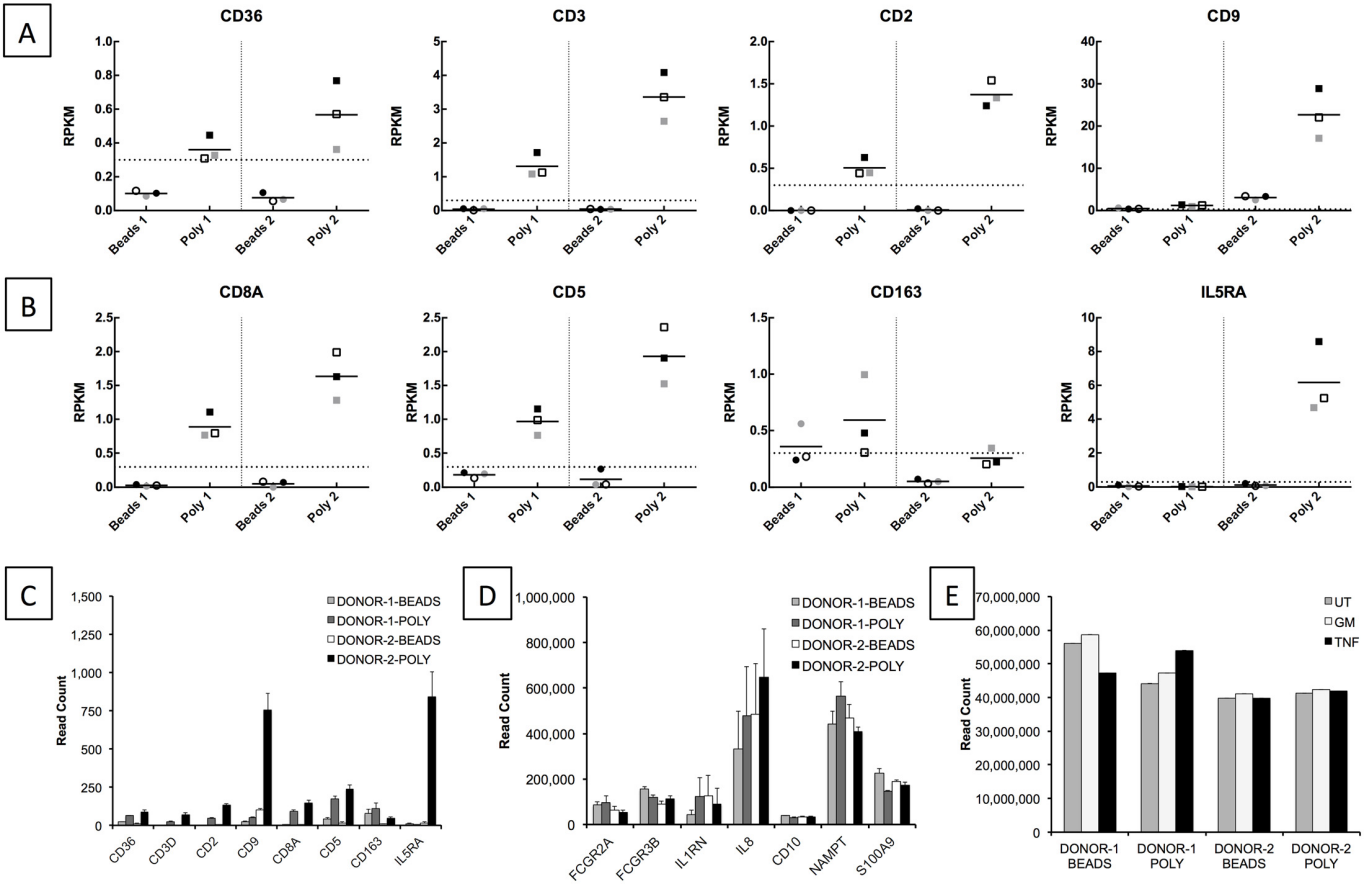
Expression levels of non-neutrophil genes in neutrophil preparations. RPKM values for non-neutrophil genes of the antigen targets in the StemCell magnetic bead negative selection isolation kit (A) and (B) non-neutrophil specific genes associated with T and B cells, monocytes, and eosinophils. Neutrophils were either isolated by negative selection (Bead, circle) or by Polymorphprep (Poly, square) from Donor 1 (1) and Donor 2 (2). Neutrophils were treated with 5 ng/mL GM-CSF (shaded grey), 10ng/mL TNFα (shaded white) or untreated (shaded black) for 1h. Horizontal dotted lines represent RPKM expression threshold of 0.3. Horizontal bars represent mean value. (C) The number of read fragments mapping to non-neutrophil genes and (D) neutrophil-specific genes in each library. Data is shown as the average (±SEM) across three treatment conditions (UT, TNFα, GM-CSF) for each Donor and each isolation protocol. (E) The number of mapped reads in each dataset.

To quantify the efficiency of the negative selection protocol to deplete contaminating cells, transcripts for the cell-specific antigens utilised in the antibody-based negative selection kit were analysed. Of the seven antigens, only transcripts for CD9, CD36, CD2, and CD3 (expressed in eosinophils, monocytes, T-cells/NK cells and T cells, respectively) were detected in any of the samples (Fig 4A). Transcripts for CD36, CD3 and CD2 were detected in all samples from both donors in samples prepared by Polymorphprep, but were absent (or below the RPKM threshold of 0.3) from all samples prepared by negative selection. CD9 transcripts (specific to eosinophils) were highest in the untreated sample from Donor 2 after purification by Polymorphprep (RPKM = 28.9), in line with the observed high numbers of eosinophils present in these preparations. Neutrophils isolated by negative selection had much lower levels of CD9 expression than those isolated by Polymorphprep. However, unlike the other transcripts, levels of CD9 where not entirely absent in samples isolated by negative selection, and were at or around the 0.3 RPKM threshold.

The levels of transcripts encoding each of the seven negative selection kit antigen target antibodies correlate well with the overall levels of cellular contamination measured by cytospins and flow cytometry. However, whilst these gene products are known to be unique cell-surface markers on specific cell types, it is possible that they may also be transcribed in other leukocytes, but not translated and expressed. We therefore measured the expression levels of other transcripts, which, according to the literature, are only expressed in non-neutrophil leukocytes, in order to further elucidate the extent that contaminating cells contribute to the transcriptome profile of the neutrophil preparations. Fig 4B shows the RPKM levels for the T-and-B-cell specific transcripts CD8a and CD5 (respectively), the monocyte-specific transcript CD163, and the eosinophil transcript interleukin-5 Receptor Alpha (IL5RA). All transcripts were present at very low levels in all samples, apart from the eosinophil specific transcript IL5RA in Polymorphprep prepared samples from Donor 2 (RPKM < 10). Importantly, transcripts for “non-neutrophil” genes were at or below the gene expression threshold (RPKM = 0.3) in negatively selected neutrophils.

In the same way that transcripts for cell-surface markers were expressed at higher levels in Polymorphprep preparations from both donors, importantly, the expression of “non-neutrophil” transcripts in the paired negatively selected neutrophils was <0.3 RPKM, with the exception of CD163 in Donor 1, which was expressed just above the threshold (RPKM = 0.36), suggesting that most contaminating cells were removed. These data indicate that the major source of cellular contamination present in the Polymorphprep preparations is eosinophils and that the negative selection isolation procedure removes this contamination. Additionally, these data show that the neutrophil transcriptome is minimally affected by contamination from the small number of non-neutrophil cells present after Polymorphprep separation. Importantly, none of the transcripts from contaminating cells were affected by cytokine stimulation with TNFα or GM-CSF, and none of the genes were identified as being significantly up- or down-regulated after cytokine stimulation.

The actual number of individual reads mapping to genes from contaminating cells was extremely low across all samples. Fig 4C shows the raw read count (not adjusted for the length of the gene or the library size) for each of the “non-neutrophil” cell markers (average number of reads in UT, GM-CSF and TNFα samples for each Donor and isolation method is shown). With the exception of eosinophil-specific markers CD9 and IL5RA in the Donor 2 Polymorphprep samples, the average number of reads mapping to each of the contaminating cell gene markers was just 54. This can be compared to the read counts for neutrophil-specific markers such as FCGR2A, FCGR3B, IL1RN, IL8, CD10, NAMPT and S100A9 (Fig 4D) which are in the order of tens or hundreds of thousands. The number of mapped reads in each library is shown in Fig 4E and is in excess of 40 million; a gene with 54 reads in a library of this size represents just 0.00014% of the mapped library.

### Comparison of differentially-expressed genes between isolation methods

**Fig 5:**
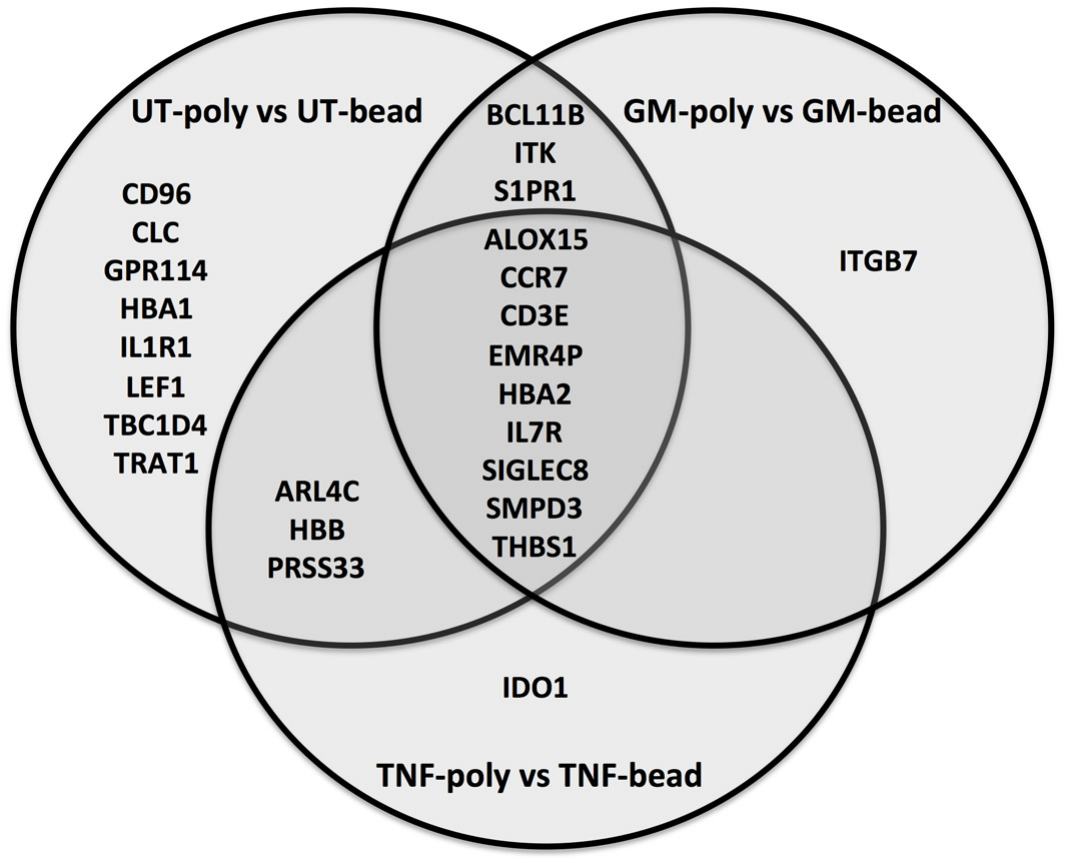
Venn diagram showing differentially expressed genes between neutrophil samples prepared by either Polymorphprep™ (poly) or magnetic beads (bead). Comparisons performed by Cufflinks using treatment specific paired-samples from two biological replicates. All genes displayed were significantly differentially expressed due to a higher RPKM in Polymorphprep prepared samples. Significance was calculated by Cuffdiff and adjusted for 5% false discovery rate by Benjamini-Hochberg correction for multiple-testing.

We performed differential expression (DE) analysis on the RNA-seq data using Cufflinks[20]. The transcriptomes from Donor 1 and Donor 2 were input as n = 2 biological replicates to compare DE in Polymorphprep and negatively selected neutrophils under the three experimental conditions (untreated, GM-CSF or TNFα for 1h). This analysis identified only 25 genes whose expression was significantly DE across all samples, representing just 0.1% of annotated genes. Of these, 23 genes were significantly DE in the untreated samples, and 9 genes were significantly DE in all three treatment pairings (Fig 5 and S1 Table). Only 2 genes in the treatment group samples (1 each in the paired GM-CSF-treated and TNFα-treated incubations) were significantly DE. In GM-CSF-treated neutrophils, *ITGB7* was expressed at higher levels in Polymorphprep isolated neutrophils (RPKM 1.55 compared to RPKM <0.3 with negative selection), and in TNFα-treated neutrophils, *IDO1* was expressed at higher levels in Polymorphprep isolated neutrophils (RPKM = 6.64 compared to RPKM<0.3 with negative selection). The greatest discrepancy in expression levels between the two isolation methods was for *CLC*, which is expressed at high levels in eosinophils. Genes encoding the α- and β- subunits of haemoglobin (*HBA1*, *HBA2* and *HBB*) were also detected in the Polymorphprep samples.

**Fig 6:**
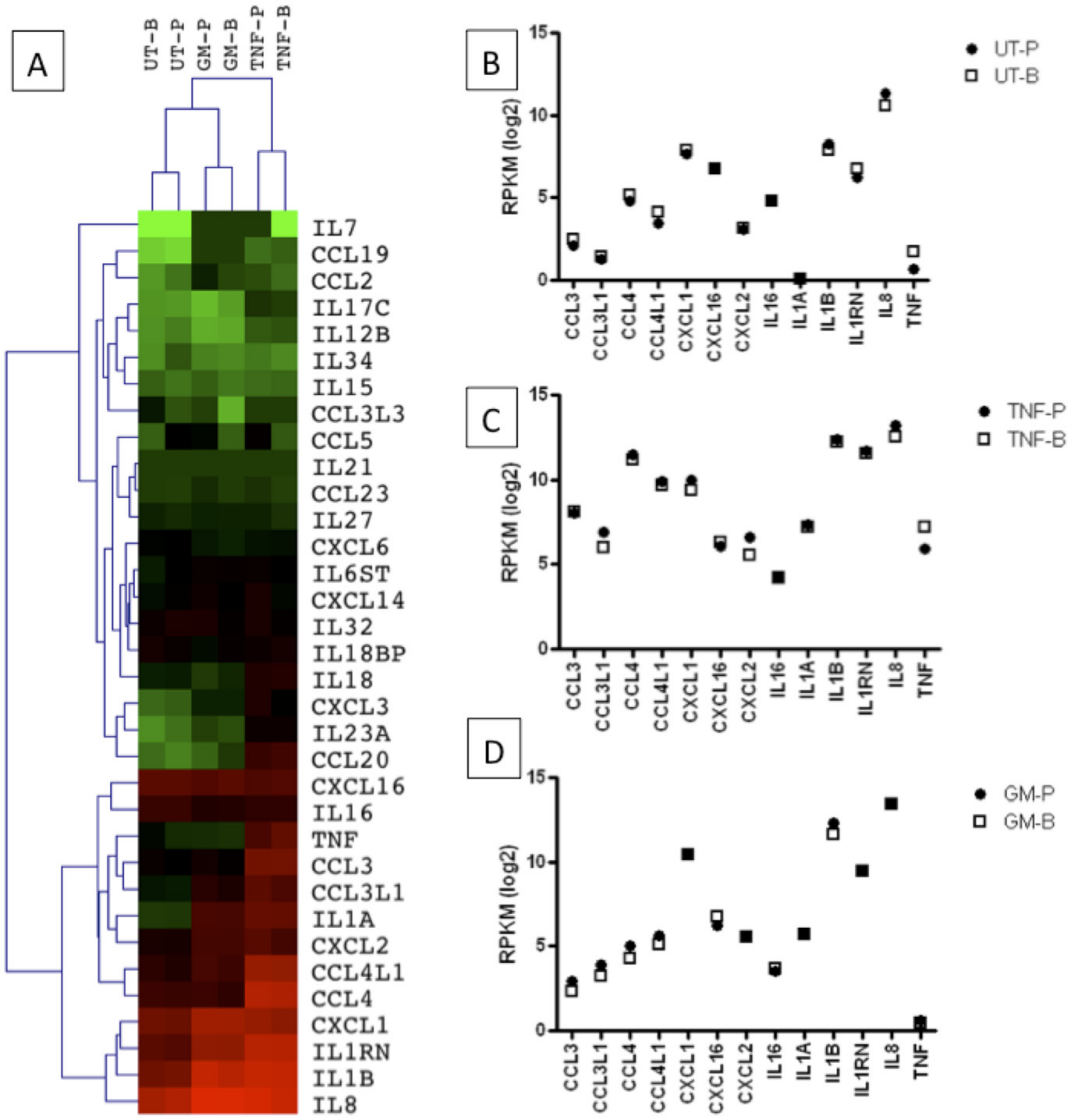
Expression of chemokine and cytokine transcripts in neutrophils isolated by Polymorphprep (-P) or negative-selection by magnetic beads (-B). (A) Cluster analysis showing expression levels (Log2 RPKM) in neutrophils incubated alone or in the presence of GM-CSF (GM) or TNF. (B-D) Expression levels of chemokine/cytokine genes with the highest level of expression in (B) untreated, (C) TNF and (D) GM-CSF treated neutrophils isolated by Polymorphprep (●) or negative selection (□).

Reports of cytokine and chemokine expression by human neutrophils can be highly dependent upon the purity of neutrophils, which may explain conflicting reports on the expression of, for example, IL-6 and IL-17A by human neutrophils[1,21,22]. We further analysed the transcriptome data of cytokine-treated (GM-CSF, TNFα) neutrophils isolated by Polymorphprep and negative selection, and compared the expression of transcripts for cytokines (interleukins and TNF-family) and chemokines (CCL, CXCL families). Importantly, none of the transcripts were significantly DE in paired Polymorphprep and negative selection samples. We performed cluster analysis of the expression levels of those transcripts that had detectable expression (RPKM>0.3) in at least one dataset (Fig 6A). A breakdown of RPKM values for cytokines with the highest levels of expression across all datasets (lower cluster) can be seen in Fig 6B–6D.

## Discussion

Despite recognition of the importance of neutrophil purity for *in vitro* studies, very few investigations have focused on the impact of purity or isolation method on neutrophil behaviour. In this study, peripheral blood neutrophils from two healthy controls were prepared using two different isolation methods (density gradient centrifugation on Polymorphprep and antibody-based negative selection). The two donors were selected because previous work in our laboratories had identified these as having low levels (Donor 1) or high levels (Donor 2) of contaminating cells, which were mainly eosinophils. Neutrophil purity following each isolation protocol was broadly in line with previously published data[6,11,21,23–25]. In contrast to the increased neutrophil purity following negative selection, the absolute yield of neutrophils obtained from whole blood was significantly lower than using Polymorphprep or Ficoll-Paque. This difference in yield raises considerable concerns for neutrophil studies where large numbers of cells are required (for example RNA studies across a range of time points). Moreover, in situations where the available volume of whole blood is restricted, for example in neutropenic or paediatric patients, this method of neutrophil purification may be unworkable. Apart from the decreased yield of neutrophils obtained by the negative selection method, it is also important to determine if this method selects for a particular sub-population of blood neutrophils, which may have different molecular or functional properties to those which are “lost” during this protocol.

RNA-Seq analysis of neutrophil samples revealed that transcripts for contaminating cells were broadly in line with expected contamination levels measured by flow cytometry and microscopic analysis. Eosinophil transcripts (CD9, IL5RA and CCL2) were at highest levels in Donor 2 Polymorphprep samples, with CD9 being the highest non-neutrophil transcript. Interestingly, despite its use as an eosinophil-specific cell surface antigen in the negative selection protocol, neutrophils are reported to express CD9 on their cell surface[26], albeit under disease conditions. This expression could therefore compromise the efficacy of the negative selection protocol in experiments isolating neutrophils from patients with inflammatory diseases. While it is commonly accepted that negative selection provides more highly-pure neutrophil suspensions, our experiments have revealed that transcripts unique to contaminating cells are still detectable, albeit at very low levels, within the negatively-selected samples. Of note, levels of the monocyte marker, CD163 were higher in negative selection samples from Donor 1 than in Polymorphprep isolated samples from Donor 2, highlighting the importance and effect of donor variation on the purity of neutrophils irrespective of the isolation method[27].

Our experiments showed that neutrophil suspensions isolated by Polymorphprep express only very low levels of transcripts that are attributable to contaminating leukocytes. Furthermore, cytokine treatment had very little effect on the number of genes differentially expressed in the samples prepared by the different isolation methods. We found that only a small number of genes were significantly differentially expressed in samples prepared by the two isolation methods using datasets for both donors. Importantly, when comparing cytokine-treated samples obtained by both isolation methods there was only a single additional significant gene in each treatment group (integrin beta 7 (ITGB7) and Indoleamine 2,3-dioxygenase 1 (IDO1) for GM-CSF and TNFα samples, respectively). This indicates that any contaminating cells in these preparations, even those prepared by Polymorphprep, did not respond to these cytokines by alterations in gene expression that contributed to the overall transcriptome profile. Therefore, cytokine-regulated changes in gene expression can confidently be attributed to altered transcriptional activity in neutrophils.

The conclusions from our RNA-Seq analyses of paired Polymorphprep and negative selection-isolated neutrophils were derived from n = 2 biological replicates at a single, 1h post-cytokine-stimulation timepoint. We now have (unpublished) RNA-Seq library data from healthy and diseased human neutrophils isolated either by Polymorphprep or negative selection, and the expression levels of the non-neutrophil contaminating genes discussed in this study are not significantly different between samples, supporting the data presented in this report. Indeed, the libraries cannot be distinguished as Polymorphprep or negative selection based on the expression levels of non-neutrophil cell markers. The 1h timepoint was selected because we wanted to observe the direct effects of these cytokines on neutrophil gene expression, and avoid any changes in gene expression that may arise as a result of, for example, secondary cytokine expression. For example, it has recently been shown that agonists such as the TLR7/8 agonist R848 can induce expression of interleukin-6 in human neutrophils by a mechanism that requires chromatin remodelling and longer incubation times (>6h) that involves autocrine TNF expression and signalling[28]. Expression of interleukin-6 has been controversial in human neutrophils and has sometimes been attributed to contaminating monocytes. Interestingly, and importantly, interleukin-6 mRNA was not detected in any of the Polymorphprep or negatively selected neutrophil samples presented in this study.

In summary, whilst neutrophil purity is of significant importance for *in vitro* studies using high-sensitivity assays such as RNA-Seq or mass spectrometry, density gradient based separation protocols such as Polymorphprep provide a suitable method of isolating unprimed, viable neutrophils, with an overall purity exceeding 96%. The major contaminating cells in neutrophil samples prepared by Polymorphprep are eosinophils, and whereas negative selection is effective for increasing neutrophil purity to approximately 98% it does so at the expense of overall yield and increased costs. Notwithstanding the potential effect of a small number of contaminating cells on the functional response of neutrophils to extracellular agonists[7], which was outside the scope of this study, our data show that reliable and robust transcriptomic data of both endogenous and cytokine-regulated neutrophil gene expression can be obtained from neutrophil samples prepared by Polymorphprep, provided that the overall level of neutrophil purity is >95%.

## Supporting Information

S1 Table The 25 genes whose expression was significantly DE in paired neutrophil samples isolated by Polymorphprep and negative selection. (PDF)
