# Supplementary material for: Whose Gene Is It Anyway? The Effect of Preparation Purity on Neutrophil Transcriptome Studies
Source: PLoS One. 2015 Sep 24;10(9):e0138982. doi: 10.1371/journal.pone.0138982 (PMC4581699; doi:10.1371/journal.pone.0138982)
Supplement: S1 Table — (PDF) [file pone.0138982.s001.pdf]

Supplementary Table 1. The 25 genes whose expression was significantly DE in paired neutrophil samples isolated by Polymorphprep and negative selection.

Gene name	Polymorphprep (RPKM)	Negative Selection (beads) (RPKM)	FDR (q-value)
**Untreated**			
ALOX15	13.11	0.01	0.000
ARL4C	17.38	0.56	0.001
BCL11B	0.96	0.02	0.008
CCR7	3.06	0.15	0.016
CD3E	4.95	0.06	0.000
CD96	1.17	0.06	0.032
CLC	175.74	9.80	0.030
EMR4P	6.76	0.18	0.003
GPR114	1.41	0.02	0.009
HBA1	26.40	0.06	0.000
HBA2	26.83	0.12	0.000
HBB	14.83	0.13	0.000
IL1RL1	4.12	0.07	0.032
IL7R	6.55	0.36	0.009
ITK	1.14	0.06	0.015
LEF1	1.39	0.04	0.046
PRSS33	9.27	0.02	0.000
S1PR1	5.20	0.27	0.011
SIGLEC8	2.91	0.04	0.001
SMPD3	4.49	0.06	0.000
TBC1D4	0.39	0.01	0.048
THBS1	18.41	0.09	0.000
TRAT1	1.34	0.02	0.048
**GM-CSF**			
ALOX15	8.01	0.06	0.000
BCL11B	0.69	0.02	0.025
CCR7	2.13	0.11	0.038
CD3E	3.25	0.05	0.003
EMR4P	5.18	0.14	0.006
HBA2	19.31	0.03	0.012
IL7R	4.44	0.27	0.012
ITGB7	1.55	0.04	0.008
ITK	0.94	0.04	0.012
S1PR1	4.96	0.21	0.005
SIGLEC8	1.89	0.04	0.003
SMPD3	2.49	0.04	0.003
THBS1	4.44	0.19	0.040
**TNFα**			
ALOX15	10.35	0.05	0.000

ARL4C	9.80	0.48	0.044
CCR7	3.73	0.19	0.044
CD3E	3.67	0.06	0.012
EMR4P	5.38	0.16	0.017
HBA2	21.46	0.13	0.002
HBB	11.70	0.10	0.022
IDO1	6.64	0.25	0.025
IL7R	5.45	0.18	0.002
PRSS33	7.17	0.01	0.027
SIGLEC8	2.26	0.02	0.002
SMPD3	3.17	0.05	0.002
THBS1	17.64	0.31	0.000
